# Berberine Protects Against NLRP3 Inflammasome via Ameliorating Autophagic Impairment in MPTP-Induced Parkinson’s Disease Model

**DOI:** 10.3389/fphar.2020.618787

**Published:** 2021-01-27

**Authors:** Shuxuan Huang, Hanqun Liu, Yuwan Lin, Muchang Liu, Yanhua Li, Hengxu Mao, Zhiling Zhang, Yunlong Zhang, Panghai Ye, Liuyan Ding, Ziting Zhu, Xinling Yang, Chaojun Chen, Xiaoqin Zhu, Xiaoyun Huang, Wenyuan Guo, Pingyi Xu, Lin Lu

**Affiliations:** ^1^ Department of Neurology, The First Affiliated Hospital of Guangzhou Medical University, Guangzhou, China; ^2^ Department of Neurology, The People’s Hospital of Guangxi Zhuang Autonomous Region, Nanning, China; ^3^ Department of Medical Affair, The First Affiliated Hospital of Guangzhou Medical University, Guangzhou, China; ^4^ Department of Neuroscience, School of Basic Medical Sciences, Guangzhou Medical University, Guangzhou, China; ^5^ Department of Neurology, The Second Affiliated Hospital of Xinjiang Medical University, Urumqi, China; ^6^ Department of Neurology, Guangzhou Chinese Medical Integrated Hospital (Huadu), Guangzhou, China; ^7^ Department of Physiology, School of Basic Medical Sciences, Guangzhou Medical University, Guangzhou, China; ^8^ Department of Neurology, The affiliated Houjie Hospital, Guangdong Medical University, Dongguan, China

**Keywords:** Parkinson’s disease, NLRP3, neuroinflammation, autophagy, berberine

## Abstract

The NLR family pyrin domain containing 3 (NLRP3) inflammasome was reported to be regulated by autophagy and activated during inflammatory procession of Parkinson’s disease (PD). Berberine (BBR) is well-studied to play an important role in promoting anti-inflammatory response to mediate the autophagy activity. However, the effect of Berberine on NLRP3 inflammasome in PD and its potential mechanisms remain unclear. Hence, in this study, we investigated the effects of BBR on 1-Methyl-4-phenyl-1,2,3,6-tetrahydropyridine (MPTP)-induced PD mice, by evaluating their behavioral changes, dopaminergic (DA) neurons loss, neuroinflammation, NLRP3 inflammasome and autophagic activity. BBR was also applied in BV2 cells treated with 1-methyl-4-pehnyl-pyridine (MPP+). The autophagy inhibitor 3-Methyladenine (3-MA) was administrated to block autophagy activity both *in vivo* and *in vitro*. In our *in vivo* studies, compared to MPTP group, mice in MPTP + BBR group showed significant amelioration of behavioral disorders, mitigation of neurotoxicity and NLRP3-associated neuroinflammation, enhancement of the autophagic process in substantia nigra (SN). *In vitro*, compared to MPP+ group, BBR significantly decreased the level of NLRP3 inflammasome including the expressions of NLRP3, PYD and CARD domain containing (PYCARD), cleaved caspase 1 (CASP1), and mature interleukin 1 beta (IL1B), via enhancing autophagic activity. Furthermore, BBR treatment increased the formation of autophagosomes in MPP+-treated BV2 cells. Taken together, our data indicated that BBR prevents NLRP3 inflammasome activation and restores autophagic activity to protect DA neurons against degeneration *in vivo* and *in vitro*, suggesting that BBR may be a potential therapeutic to treat PD.

## Introduction

Parkinson’s disease (PD) is characterized by loss of dopaminergic (DA) neurons and formation of Lewy bodies in substantia nigra (SN), afflicting approximately 1% of the population aged 60 years and older worldwide (Ascherio and Schwarzschild, 2016). At present, there is no radical therapy for PD (Ryan et al., 2019), and it is necessary to elucidate the underlying mechanism of PD to develop novel therapeutic methods. Numerous studies have demonstrated that NLR family pyrin domain containing 3 (NLRP3) inflammasome plays a vital role in the pathogenesis of PD (Haque et al., 2020). The activation of NLRP3 inflammasome triggered by toxins leads to the cleavage of caspase 1 (CASP1) into cleaved CASP1, which results in the secretion of interleukin 1 beta (IL1B) and interleukin 18 (IL18) to induce neuroinflammation and neuron death (Heneka et al., 2018).

NLRP3 inflammasome accumulates in microglia of 1-methyl-4-phenyl-1,2,3,6- tetrahydropyridine (MPTP)-induced mice and leads to DA neurons loss (Lee et al., 2019). 1-methyl-4-pehnyl-pyridine (MPP+), a toxic metabolite of MPTP, has been used as a stimulant to mimic PD pathophysiology *in vitro*. Recent studies have also reported that MPP+ can activate NLRP3 inflammasome in microglia (Yao et al., 2019; Zeng et al., 2019; Cheng et al., 2020). Therefore, inhibition of NLRP3 inflammasome activation may be a critical strategy to alleviate PD neuroinflammation.

Autophagy is an evolutionary homeostatic cellular process to degrade damaged organelles and harmful proteins. The multi-step process of autophagy initiated and mediated by a series of autophagy related (Atg) genes such as beclin 1 (BECN1) and microtubule associated protein 1 light chain 3 beta (MAP1LC3B) (Lu et al., 2019; Pohl and Dikic, 2019). Studies have shown that autophagy activation could ameliorate the detrimental effects of neuroinflammation, and thereby protect against chronic inflammatory in PD (Menzies et al., 2017; Ali et al., 2020). Although multiple evidences revealed that autophagy regulates NLRP3 inflammasome thus mitigating inflammatory response (Han et al., 2019; Houtman et al., 2019; Mehto et al., 2019; Fei et al., 2020), although the underlying mechanism of how autophagy affects the activation of NLRP3 inflammasome in PD is not completely understood. Hence, the inhibition of NLRP3 inflammasome via autophagic enhancement may be a potential benefit of PD therapy. Berberine (BBR), an organic isoquinoline alkaloid, has been clinically used in the treatment of various diseases such as cancer, bacterial diarrhea, type 2 diabetes, hypercholesterolemia, inflammation, and cardiac diseases (Neag et al., 2018; Belwal et al., 2020; Song et al., 2020). However, the neuroprotective efficacy and underlying mechanism of BBR in PD remains elusive. Therefore, we implemented MPTP-induced PD *in-vivo* model and MPP+-induced *in-vitro* model, to investigate the neuroprotective and anti-neuroinflammatory effects of BBR in ameliorating PD-like symptoms and to elucidate the role of autophagy in ameliorating neuroinflammation in PD.

## Materials and Methods

### Animals

Eight-weeks-old C57BL/6J male mice (24–31 g) were ordered from Guangdong Medical Experimental Animal Center and housed in a controlled environment in terms of temperature, humidity, and a 12/12-h light/dark cycle, along with food and water ad libitum. All the procedures were performed in compliance with the Institute’s guidelines and the Guide for the Care and Use of Laboratory Animals. The study was approved by the institutional animal care committee of Guangzhou Medical University.

### *In-vivo* Experimental Design and Drug Treatments

**FIGURE 1:**
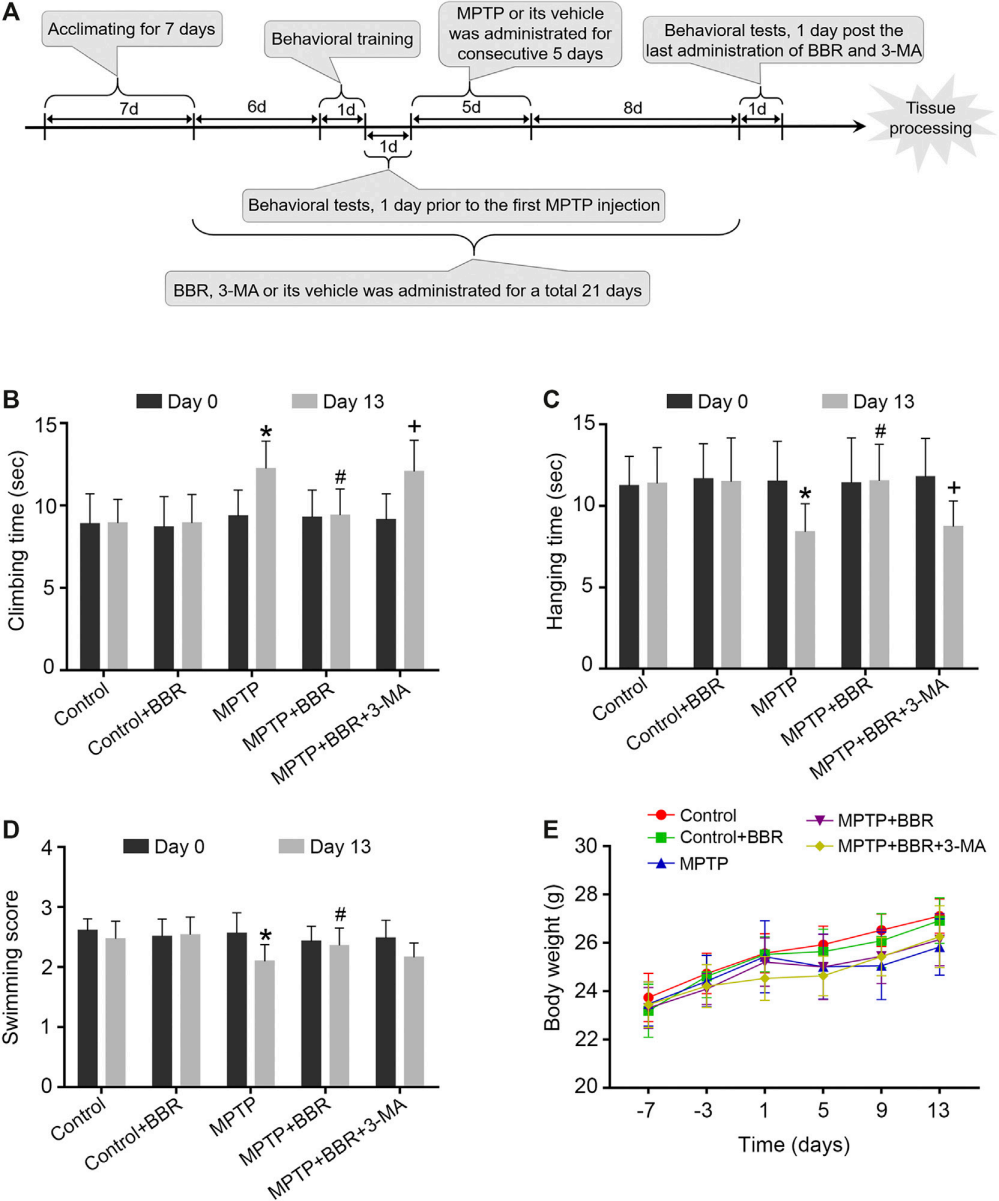
The flowchart of the experimental procedure and neurobehavioral tests in MPTP-induced mice. **(A)** The flowchart of the experimental procedure. **(B)** Time spent in climbing of the pole test. **(C)** Time spent in hanging on the line of the hanging test. **(D)** Swimming scores in the swimming test. **(E)** Body-weight changes at different time points. Data were expressed as the mean ± SD (*n* = 12). *p < 0.05 compared with control group, ^#^ *p* < 0.05 compared with MPTP group, ^+^ *p* < 0.05 compared with MPTP + BBR group.

Mice were randomly divided into five groups as control, control + BBR, MPTP, MPTP + BBR, and MPTP + BBR + 3-Methyladenine (3-MA) group, respectively (*N* = 12 per group). BBR, MPTP, and 3-MA were purchased from Sigma-Aldrich Ltd. (Sigma, United States, PHR1502, M0896, M9281, respectively) and dissolved in 0.9% saline. The certified purity of BBR was 88.4%. Prior to MPTP injection for 7 days, 50 mg/kg BBR was intragastrically administrated to mice in control + BBR, MPTP + BBR, and MPTP + BBR + 3-MA groups once daily for 21 days, and the same volume of 0.9% saline was intragastrically administered to the control and MPTP groups. For the mice from MPTP, MPTP + BBR, and MPTP + BBR + 3-MA groups, after one week’s 0.9% saline or BBR treatment, 30 mg/kg MPTP was subcutaneously injected once a day for 5 consecutive days to establish the MPTP-induced subacute PD model, and the same volume of 0.9% saline were subcutaneously administered to the control and control + BBR groups. In addition, 5 mg/kg 3-MA was intraperitoneally administered in MPTP + BBR + 3-MA group once a day for 21 days. Simultaneously, the same volume of 0.9% saline was intraperitoneally administered to the control, control + BBR, MPTP, and MPTP + BBR groups. BBR and 3-MA were administrated in the same day whereas BBR and 3-MA were injected at 8:00 am and 4:00 pm, respectively. The animal behavioral tests including pole, hanging, and swimming tests were performed one day before MPTP injection and the last day after BBR and/or 3-MA treatment separately. All behavioral tests on each mouse were conducted three times at 10-min intervals, and the observer was blinded to all animals. Besides, the body weight of mice was recorded once every 4 days. The timeline of the experimental procedure was shown in Figure 1A.

### Pole Test

A pole of 50 cm in length and 1 cm in diameter was set upright and a wooden ball wrapped with gauze was adhered to the top of the pole. Mice were placed on the top of the wooden ball and the time until they reached the bottom of the pole was recorded. The average time of three trials was recorded.

### Hanging Test

A horizontal wire of 1.5 mm in diameter was suspended 30 cm above a foam carpet. Each mouse was forced to grip the wire with its forelimbs and its hanging time was recorded until the mouse fell onto the foam carpet. The average time of three trials was recorded.

### Swimming Test

A container of water (dimensions, 20×30 × 20 cm) was used for swimming test. The depth of the water was 10 cm and the temperature was 22–25 °C. Each mouse was forced to swim for one minute and the swimming scores were determined according to a previous study (Donnan et al., 1987). Briefly, scoring was based on the following: continuous swimming movements = 3; occasional floating = 2.5; floating > 50% of the time = 2.0; occasional swimming only = 1.5; occasional swimming using hind limbs while floating on side = 1.0; and no use of limbs = 0. The average time of three trials was recorded.

### Tissue Preparation

Following the completion of the last behavioral tests, mice were anesthetized, and then transcardially perfused with 4% paraformaldehyde in 0.1 M of phosphate-buffered saline (PBS). The brains were post-fixed in 4% paraformaldehyde overnight at 4 °C, then gradually transferred to 10%, 20%, and 30% (w/v) sucrose solutions for cryoprotection. Coronal tissue blocks were cut into 10 µm thickness of sections (Leica CM1950, Heidelberg). The spanning blocks of tissue region were in 0.74 to 0.26 mm from bregma for striatum and −2.92 to −3.64 mm from bregma for SN. Sections were thaw-mounted to adhesive microscope slides and stored at −80 °C.

### Immunohistochemistry

For immunohistochemical analysis, brain sections were incubated with 3% H_2_O_2_ for 20 min to block the activity of endogenous peroxidases. After incubating with primary anti-tyrosine hydroxylase (TH) (1:500; Santa Cruz, sc-25269), anti-PYD and CARD domain containing (PYCARD) (1:200; Immunoway, T0365), anti-IL1B (1:200; ABclonal, A13268), and anti-MAP1LC3B (1:1,000; ABclonal, A11282) antibodies overnight at 4 °C, sections were incubated with the Two-step Plus Poly-horseradish peroxidase (HRP) Anti-Mouse/Rabbit IgG Detection System (Dako, United States). Finally the tissue sections were treated with 3,3′-diaminobenzidine and hematoxylin. The optical densities (OD) of TH in striatum was calculated by the ImageJ software. The positive cells of TH, MAP1LC3B, PYCARD, and IL1B in SN were manually counted by researchers blinded to the treatment groups.

The immunofluorescence without block of endogenous peroxidases was examined with incubation of primary anti-TH (1:500; Santa Cruz, sc-25269), anti-allograft inflammatory factor 1 (AIF1) (1:200; Abcam, ab178874), anti-glial fibrillary acidic protein (GFAP) (1:200; Abcam, ab7260), anti-NLRP3 (1:200; AdipoGen, AG-20B-0014-C100), anti-PYCARD (1:200; Immunoway, T0365), anti-CASP1 (1:200; ABclonal, A0964) and anti-MAP1LC3B (1:1,000; ABclonal, A11282) antibodies overnight at 4 °C. Sections were incubated with Alexa Fluor 568-conjugated goat anti-rabbit IgG (1:1,000; Abcam, ab175471) and Alexa Fluor 488-conjugated goat anti-mouse IgG (1:1,000; Invitrogen, A32723) for 1 h at room temperature. After washing with 0.01 M PBS, sections were stained with 4′,6-Diamidino-2′-phenylindole (DAPI) (Sigma, United States) for nuclear staining, and visualized under fluorescent microscope. GFAP and AIF1 positive cells in SN were manually counted by researchers blinded to the treatment groups. The mean fluorescence intensity (MFI) of NLRP3, PYCARD, and CASP1 were calculated by the ImageJ software. The MAP1LC3B puncta were manually counted by researchers blinded to the treatment groups.

### Nissl Staining

Nissl staining was performed according to the manufacturer’s instructions of nissl staining solution (Beyotime, Shanghai, China). The positive cells were viewed under a microscope. The number of neurons in SN was manually counted by researchers blinded to the treatment groups.

### Transmission Electron Microscopy

For analysis of autophagosome by transmission electron microscopy, mouse SN tissues were cut into a size of 0.5–1.0 mm^3^ and post-fixed with 2.5% glutaraldehyde overnight at 4 °C. These blocks were washed three times with 0.1 M PBS, and post-fixed in 1% osmium tetroxide for 2 h at 4 °C. The blocks were microdissected to ultrathin sections (60–70 nm), post-stained with uranyl acetate and lead citrate, then examined under an electron microscope (Philips, Amsterdam, Netherlands).

### Quantitative Polymerase Chain Reaction

Total RNA was extracted by using Trizol reagent (Invitrogen, United States) following the manufacturer’s instructions. cDNA was synthesized using PrimeScipt RT Master Mix (Takara, Japan). qPCR was performed on a Bio-rad Cx96 Detection System (Bio-rad, United States) by using a SYBR green PCR kit (Applied Biosystems, United States). The primers for the targeted genes were shown in Table 1. Reaction conditions were 95 °C for 5 min, followed by 40 cycles of 95 °C for 15 s and 60 °C for 1 min mRNA quantification was normalized to ACTB as an internal standard.

**TABLE 1:** Primer sequences used for the qPCR analysis.

Gene name	Forward primer sequence (5′-3′)	Reverse primer sequence (5′-3′)
NLRP3	ATT​ACC​CGC​CCG​AGA​AAG​G	TCG​CAG​CAA​AGA​TCC​ACA​CAG
IL1B	GAA​ATG​CCA​CCT​TTT​GAC​AGT​G	TGG​ATG​CTC​TCA​TCA​GGA​CAG
ACTB	GGC​TGT​ATT​CCC​CTC​CAT​CG	CCA​GTT​GGT​AAC​AAT​GCC​ATG​T

### Western Blotting Analysis

For western blotting analysis, total proteins were collected from striatum, SN, and BV2 cells and stored at −80 °C. 40 ug of total protein lysate was loaded onto a 12% sodium-dodecyl-sulfate polyacrylamide gel in each lane, then transferred onto a polyvinylidene-difluoride membrane (Millipore, United Ststes). The primary antibodies were incubated overnight at 4 °C included anti-TH (1:500; Santa Cruz, sc-25269), anti-solute carrier family 6 member 3 (SLC6A3) (1:1,000; ABclonal, A152360), anti-dopamine receptor D2 (DRD2) (1:1,000; ABclonal, A12930), anti-AIF1 (1:1,000; Santa Cruz, sc-32725), anti-GFAP (1:1,000; ABclonal, A14673), anti-NLRP3 (1:200; AdipoGen, AG-20B-0014-C100), anti-PYCARD (1:1,000; Immunoway, T0365), anti-CASP1 (1:1,000; ABclonal, A0964), anti-IL1B (1:1,000; ABclonal, A12688), anti-MAP1LC3B (1:1,000; ABclonal, A11282), anti-BECN1 (1:1,000; Cell Signaling Technology, 3738S) and anti-ACTB (1:3,000; ABclonal, AC026). HRP-conjugated anti-Rabbit antibody (1:5,000; ABclonal, AS014) or anti-Mouse antibody (1:5,000; ABclonal, AS003) was used as secondary antibody. ImageJ software was used to quantify the target bands and ACTB as an internal control.

### *In-vitro* Experiments

BV2 cells were cultured in Dulbecco’s modified eagle medium (HyClone) supplemented with 10% fetal bovine serum (Gibco) and 100 U/ml penicillin (Invitrogen) at 37 °C in a humidified atmosphere with 5% CO_2_. Cells were treated with MPP+ at 0, 10, 50, 100, 200, and 400 μM concentrations for 24 hours to detect the cells cytotoxicity. Cells treated with BBR at 0, 12.5, 25, 50, 100, and 200 μM concentrations for 24 h were conducted for CCK8 assays. Based on the two batches results, for MPP+ group, cells were treated with MPP+ at 200 μM for 24 h. For MPP+ + BBR groups, cells were incubated with BBR at 0, 12.5, 25, 50 μM concentrations for 3 h respectively, prior to be treated with MPP+ at 200 μM for 24 h. For MPP+ + BBR + 3-MA group, cells were pre-treated with BBR at 25 μM and 3-MA at 10 mM for 3 h, then were treated with 200 μM MPP+ for 24 h. For control group, cells were treated with the same volume of culture medium. All groups of cells, then were collected to examine the activation of NLRP3 inflammasome and autophagic activity.

### Enzyme-Linked Immunosorbent Assays

BV2 cells were treated with MPP+ at 0, 10, 50, 100, 200, and 400 μM concentrations for 24 h. The culture supernatants were collected and measured for IL1B via ELISA kits (Invitrogen, United States, BMS6002) according to the manufacturer’s instructions. Briefly, supernatants were added in the coated wells with IL1B antibody of 96-well plates and incubated for 2 h at room temperature, then washed five times and incubated with an HRP-linked streptavidin solution for 30 min at room temperature. All samples were tested by duplication, and absorbance at 450 nm was measured by a microplate spectrophotometer (Thermo Scientific, United Ststes).

### Monodansylcadaverine (MDC) Staining

MDC, a fluorescent marker of autophagic vacuoles, was used to detect the autophagic activity. BV2 cells were pre-treated with 25 μM BBR with or without 10 mM 3-MA for 3 h, this was followed by addition of 200 μM MPP+ for 24 h. At the end of the incubation period, 50 mM MDC (Sigma, United States, D4008) was added to the cells for 15 min at 37 °C in dark. After washing twice with 0.01 M PBS, cells were examined under a fluorescent microscope (Leica, Solms, Germany). The number of autophagic vacuoles was manually counted by researchers blinded to the treatment groups.

### Statistical Analysis

Data were presented as means ± standard deviations (SDs) and analyzed via SPSS 21.0 software. The differences among groups were compared using one-way analyses of variance (ANOVAs) followed by Tukey’s tests for post-hoc comparisons. The differences were established to be statistically significant at *p* < 0.05.

## Results

### BBR Ameliorates Behavioral Impairments in MPTP-Induced Mice

Compared to the control group, MPTP-induced mice spent significantly longer time in the pole test (*p* < 0.05, Figure 1B), shorter time in the hanging test (*p* < 0.05, Figure 1C), and achieved lower scores in the swimming test (*p* < 0.05, Figure 1D). MPTP + BBR-treated mice showed significantly better performance in behavioral tests including pole test (*p* < 0.05, Figure 1B), hanging test (*p* < 0.05, Figure 1C) and swimming test (*p* < 0.05, Figure 1D) when comparing to MPTP group. While comparing to MPTP + BBR group, mice in MPTP + BBR + 3-MA group showed significantly inferior performance in pole test (*p* < 0.05, Figure 1B) and hanging test (*p* < 0.05, Figure 1C). Compared to the control group, mice in the other four groups all showed no significant difference in body weight at all time points (*p* > 0.05, Figure 1E).

### BBR Mitigates Neurotoxicity in MPTP-Induced Mice

**FIGURE 2:**
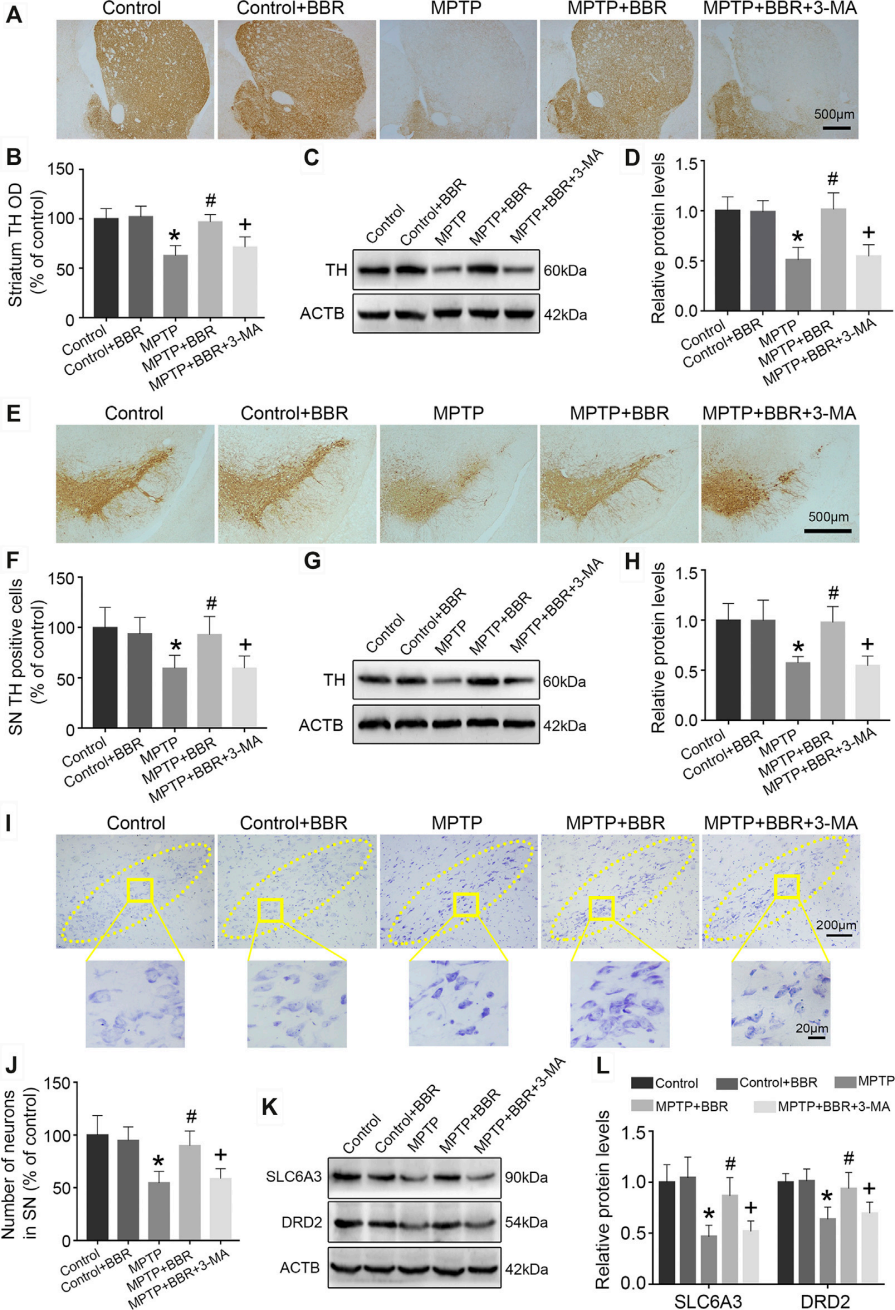
BBR ameliorates dopaminergic neurons degeneration in MPTP-induced mice. **(A)** The representative immunohistochemical staining of TH in striatum. **(B)** The OD of TH staining in striatum. Representative western blot bands **(C)** and the statistical graph **(D)** of TH in striatum. **(E)** The representative immunohistochemical staining of TH in SN. **(F)** The number of TH positive neurons in SN. Representative western blot bands **(G)** and the statistical graph **(H)** of TH in SN. **(I)** Nissl staining for neurons in SN. **(J)** The number of neurons in SN. Representative western blot bands **(K)** and the statistical graph **(L)** of SLC6A3 and DRD2 in striatum. Data were expressed as the mean ± SD (*n* = 6). *p < 0.05 compared with control group, ^#^ *p* < 0.05 compared with MPTP group, ^+^ *p* < 0.05 compared with MPTP + BBR group. TH, tyrosine hydroxylase; OD, optical densities; SN, substantia nigra; SLC6A3, solute carrier family 6 member 3; DRD2, dopamine receptor D2.

Compared to the control group, MPTP-induced mice showed significant loss of TH in striatum (*p* < 0.05, Figures 2A–D) and SN (*p* < 0.05, Figures 2E–H). Compared to the MPTP group, MPTP + BBR-treated mice showed significant increase of TH in striatum (*p* < 0.05, Figures 2A–D) and SN (*p* < 0.05, Figures 2E–H). Additionally, mice in MPTP + BBR + 3-MA group showed significant loss of TH both in striatum and SN when compared to those in MPTP + BBR group (*p* < 0.05, Figures 2A–H). As shown in Figures 2I,J, the number of DA neurons in SN in MPTP and MPTP + BBR + 3-MA groups were decreased by nissl staining (*p* < 0.05), whereas the number of DA neurons in MPTP + BBR group were increased (*p* < 0.05). Additionally, compared to control group, MPTP-induced mice showed lower expression of SLC6A3 and DRD2 in striatum (both *p* < 0.05, Figures 2K,L). MPTP + BBR-treated mice showed significant increase of SLC6A3 and DRD2 expressions in striatum when comparing to MPTP group (both *p* < 0.05, Figures 2K,L). However, mice in MPTP + BBR + 3-MA group showed significant reduction of SLC6A3 and DRD2 expression in striatum when compared with MPTP + BBR group (both *p* < 0.05, Figures 2K,L).

### BBR Ameliorates NLRP3-Associated Neuroinflammation in SN of MPTP-Induced Mice

**FIGURE 3:**
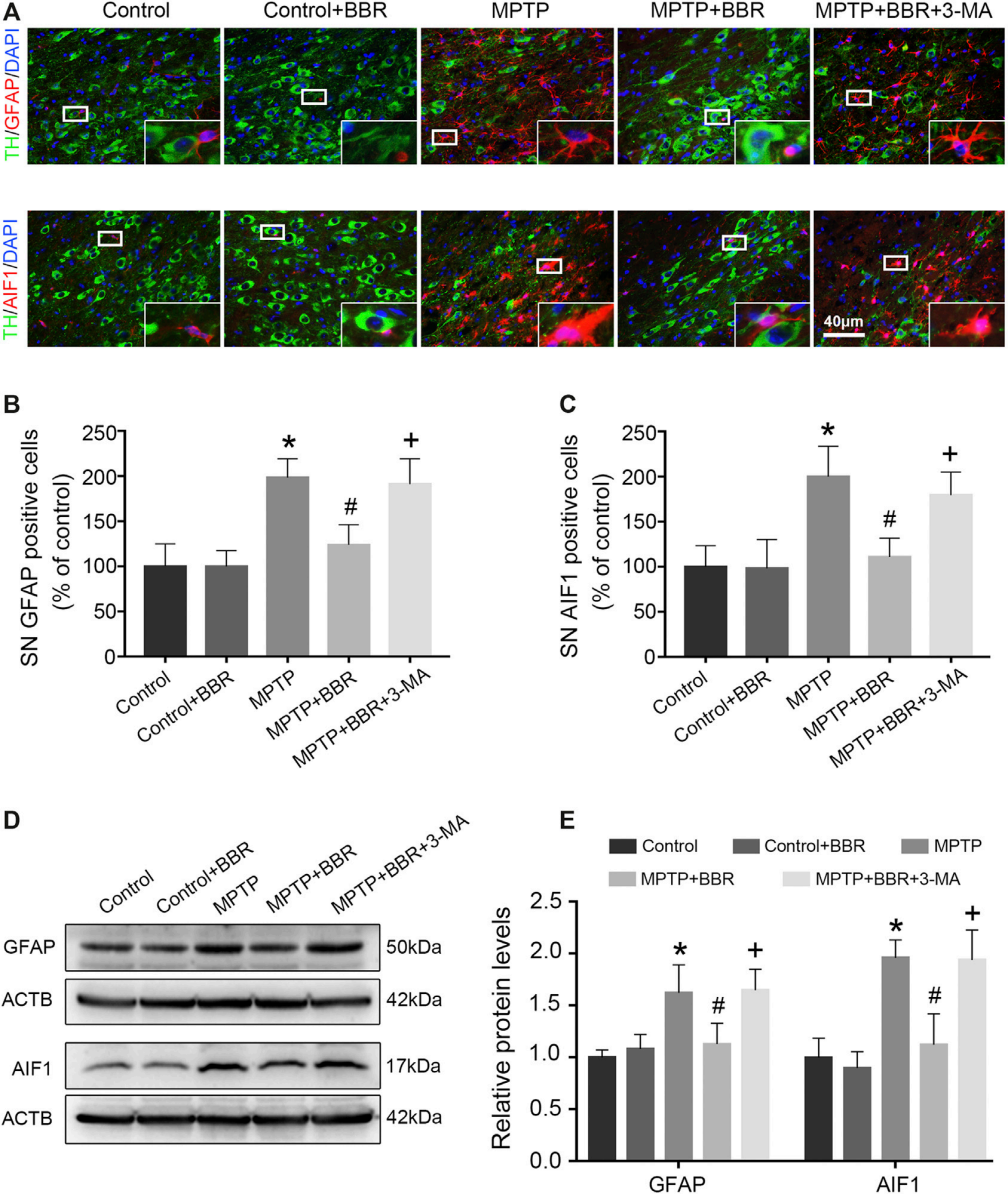
BBR inhibits MPTP-induced neuroinflammation in mice. **(A)** The representative double-immunofluorescent staining of GFAP (red) /TH (green) and AIF1 (red) /TH (green) in SN. The number of GFAP **(B)** and AIF1 **(C)** positive cells in SN. Representative western blot bands **(D)** and the statistical graph **(E)** of GFAP and AIF1 in SN. Data were expressed as the mean ± SD (*n* = 6). *p < 0.05 compared with control group, ^#^ *p* < 0.05 compared with MPTP group, ^+^ *p* < 0.05 compared with MPTP + BBR group. GFAP, glial fibrillary acidic protein; TH, tyrosine hydroxylase; AIF1, allograft inflammatory factor 1; SN, substantia nigra.

As shown in Figures 3A–C, the representative images and statistical graphs of immunofluorescent staining showed that the expressions of AIF1 and GFAP were increased in MPTP and MPTP + BBR + 3-MA groups but not in control, control + BBR and MPTP + BBR groups (both *p* < 0.05), which indicated the infiltration of microglia and astrocytes in SN of mice from MPTP and MPTP + BBR + 3-MA groups. In accordance, compared to the control group, mice in MPTP + BBR group exhibited a significant reduction in the expression of AIF1 and GFAP in SN (both *p* < 0.05, Figures 3D,E). Compared to the MPTP group, MPTP + BBR-treated mice exhibited significantly lower expression of AIF1 and GFAP in SN (both *p* < 0.05, Figures 3D,E), whereas mice in MPTP + BBR + 3-MA group significantly increased the expression of AIF1 and GFAP when compared to those in MPTP + BBR group (both *p* < 0.05, Figures 3D,E).

**FIGURE 4:**
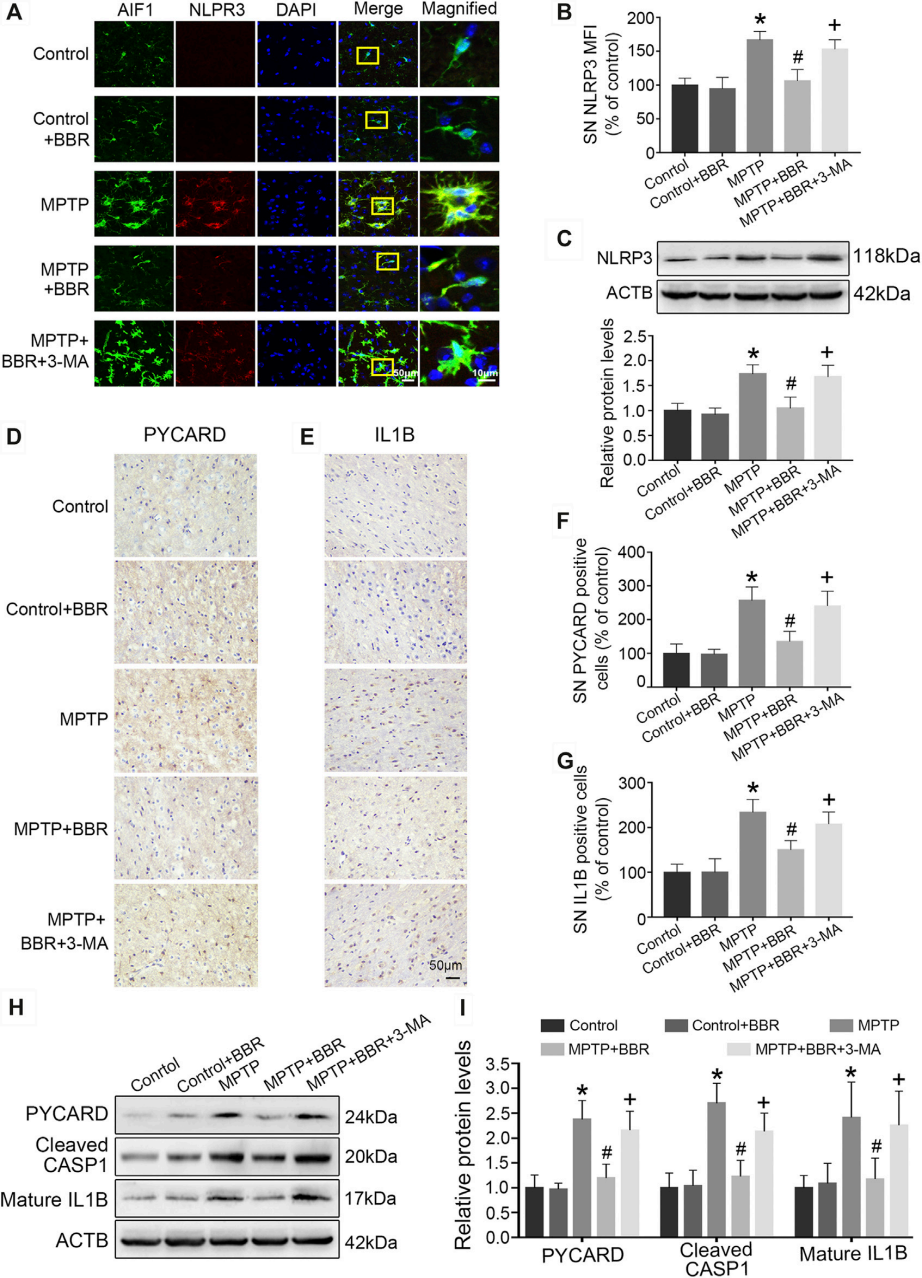
BBR suppresses NLRP3 inflammasome activation in MPTP-induced mice. **(A)** The representative double-immunofluorescent staining of NLRP3 (red) and AIF1 (green) in SN. **(B)** The MFI of NLRP3 in SN. **(C)** Representative western blot bands and the statistical graph of NLRP3 in SN. The representative immunohistochemical staining for PYCARD **(D)** and IL1B **(E)** in SN. The number of PYCARD **(F)** and IL1B **(G)** positive cells in SN. Representative western blot bands **(H)** and the statistical graph **(I)** of PYCARD, cleaved CASP1, and mature IL1B in SN. Data were expressed as the mean ± SD (*n* = 4 for Figure 4B; *n* = 6 for Figures 4C,F,G,I). *p < 0.05 compared with control group, ^#^ *p* < 0.05 compared with MPTP group, ^+^ *p* < 0.05 compared with MPTP + BBR group. NLRP3, NLR family pyrin domain containing 3; AIF1, allograft inflammatory factor 1; SN, substantia nigra; MFI, mean fluorescence intensity; PYCARD, PYD, and CARD domain containing; IL1B, interleukin 1 beta; CASP1, caspase 1.

As shown in Figure 4A, co-immunostaining revealed NLRP3 and AIF1 were almost overlapping, indicating NLRP3 mainly expressed in microglia of MPTP-induced mice. Statistical graphs of immunofluorescent staining showed that the expressions of NLRP3 were increased in MPTP and MPTP + BBR + 3-MA groups but not in control, control + BBR and MPTP + BBR groups (*p* < 0.05, Figure 4B). Compared to control group, MPTP-induced mice showed significantly higher expression of NLRP3 in SN (*p* < 0.05, Figure 4C). Compared to MPTP group, MPTP + BBR-treated mice showed significantly lower expression of NLRP3 (*p* < 0.05, Figure 4C). Additionally, mice in MPTP + BBR + 3-MA group significantly increased the expression of NLRP3 when compared to those in MPTP + BBR group (*p* < 0.05, Figure 4C). The representative images statistical graphs of immumohistochemical staining showed the increase in positive cells of PYCARD and IL1B in SN of mice from MPTP and MPTP + BBR + 3-MA groups but not in control, control + BBR and MPTP + BBR groups (both *p* < 0.05, Figures 4D–G). Furthermore, compared to control group, MPTP-induced mice exhibited significant increase in the expressions of NLRP3 inflammasome components including PYCARD, cleaved CASP1, and mature IL1B (all *p* < 0.05, Figures 4H,I). Compared to MPTP group, MPTP + BBR-treated mice showed significant decrease in the expressions of PYCARD, cleaved CASP1, and mature IL1B (all *p* < 0.05, Figures 4H,I), whereas mice in MPTP + BBR + 3-MA group significantly increased the expressions of PYCARD, cleaved CASP1, and mature IL1B when compared to MPTP + BBR group (all *p* < 0.05, Figures 4H,I).

### BBR Mitigates Autophagic Impairment in SN of MPTP-Induced Mice

**FIGURE 5:**
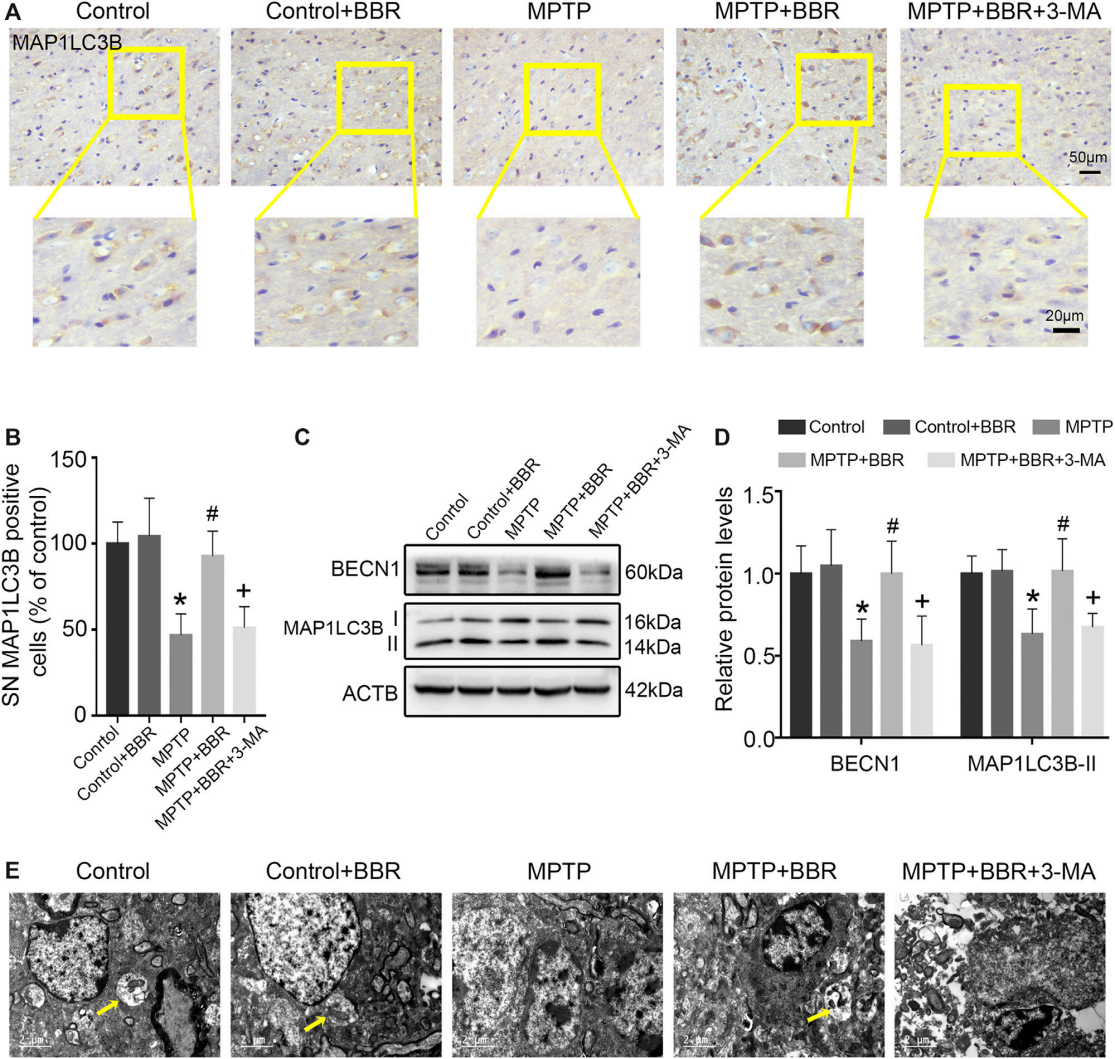
BBR mitigates autophagic impairment in MPTP-induced mice. **(A)** The representative immunohistochemical staining for MAP1LC3B in SN. **(B)** The number of MAP1LC3B positive cells in SN. Representative western blot bands **(C)** and the statistical graph **(D)** of MAP1LC3B and BECN1 in SN. **(E)** Transmission electron microscopy shown with autophagosomes (yellow arrows) in the SN. Data were expressed as the mean ± SD (*n* = 6). *p < 0.05 compared with control group, ^#^ *p* < 0.05 compared with MPTP group, ^+^ *p* < 0.05 compared with MPTP + BBR group. MAP1LC3B, microtubule associated protein 1 light chain 3 beta; SN, substantia nigra; BECN1, beclin 1.

As shown in Figures 5A,B, the representative images and statistical graphs of immunohistochemical staining results showed the decrease in MAP1LC3B positive cells in SN of mice from MPTP and MPTP + BBR + 3-MA groups but not from control, control + BBR and MPTP + BBR groups (*p* < 0.05). In accordance, compared to control group, MPTP-induced mice showed a significant decrease in the expression of BECN1 and MAP1LC3B-II (both *p* < 0.05, Figures 5C,D). Compared to MPTP group, MPTP + BBR-treated mice showed a significant increase in the expression of BECN1 and MAP1LC3B-II (both *p* < 0.05, Figures 5C,D), whereas mice in MPTP + BBR + 3-MA group significantly reduced the expression of BECN1 and MAP1LC3B-II when compared to MPTP + BBR group (both *p* < 0.05, Figures 5C,D). The representative images of transmission electron microscopy showed the formation of autophagosome in SN of mice from control, control + BBR, and MPTP + BBR groups but not from MPTP and MPTP + BBR + 3-MA groups (Figure 5E).

### BBR Decreases NLRP3 Inflammasome in MPP+-Treated BV2 Cells

**FIGURE 6:**
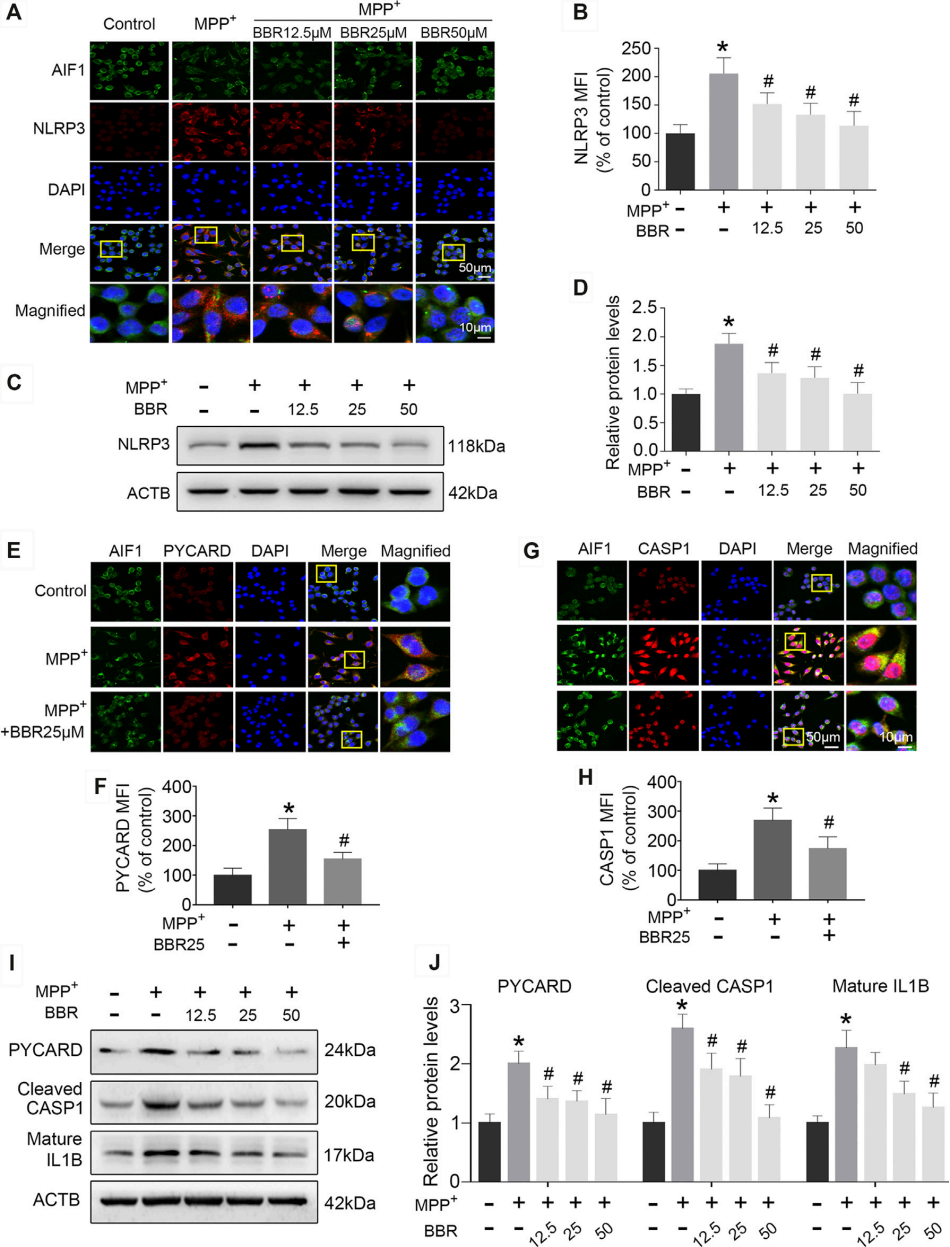
BBR inhibits NLRP3 inflammasome in MPP^+^-treated BV2 cells. The representative double-immunofluorescent staining **(A)** and MFI **(B)** of NLRP3 (red) and AIF1 (green) in BV2 cell treated with MPP^+^ at 200 μM and BBR at the concentration of 0, 12.5, 25, and 50 μM. Representative western blot bands **(C)** and the statistical graph **(D)** of NLRP3 in BV2 cells treated with MPP^+^ at 200 μM and BBR at the concentration of 0, 12.5, 25, and 50 μM. The representative double-immunofluorescent staining **(E)** and MFI **(F)** of PYCARD in BV2 cells treated with MPP^+^ at 200 μM and BBR at 25 μM. The representative double-immunofluorescent staining **(G)** and MFI **(H)** of CASP1 in BV2 cells treated with MPP^+^ at 200 μM and BBR at 25 μM. Representative western blot bands **(I)** and the statistical graph **(J)** of PYCARD, cleaved CASP1 and mature IL1B in BV2 cell treated with MPP^+^ at 200 μM and BBR at the concentration of 12.5, 25, and 50 μM. Data were expressed as the mean ± SD (*n* =3). *p < 0.05 compared with untreated group, ^#^ *p* < 0.05 compared with MPP^+^ group. NLRP3, NLR family pyrin domain containing 3; MFI, mean fluorescence intensity; AIF1, allograft inflammatory factor 1; PYCARD, PYD and CARD domain containing; CASP1, caspase 1; IL1B, interleukin 1 beta.

The representative images and statistical graphs of immunofluorescent staining showed that MPP+ at 200 μM increased the positive cells of NLRP3 in BV2 cells (*p* < 0.05), which was decreased by BBR at the concentrations of 12.5, 25, and 50 μM (all *p* < 0.05, Figures 6A,B). In accordance, compared to untreated group, MPP+ at 200 μM significantly increased the expression of NLRP3 in BV2 cells (*p* < 0.05), whereas the expression was decreased by BBR in a dose-dependent manner 12.5, 25, and 50 μM (all *p* < 0.05, Figures 6C,D). In addition, MPP+ at 200 μM significantly increased the positive cells of PYCARD (*p* < 0.05, Figures 6E,F) and CASP1 (*p* < 0.05, Figures 6G,H) in BV2 cells, which was reduced by BBR at 25 μM (both *p* < 0.05, Figures 6E–H). Furthermore, compared to untreated group, MPP+ at 200 μM significantly elevated the expressions of PYCARD, cleaved CASP1, and mature IL1B in BV2 cells (all *p* < 0.05, Figures 6I,J), whereas were reduced by BBR dose-dependently (all *p* < 0.05, Figures 6I,J). The concentrations of drugs used in BV2 cells were according to the cell survival data as shown in Supplementary Figure S1.

### BBR Enhances Autophagic Activity in MPP+-Treated BV2 Cells

**FIGURE 7:**
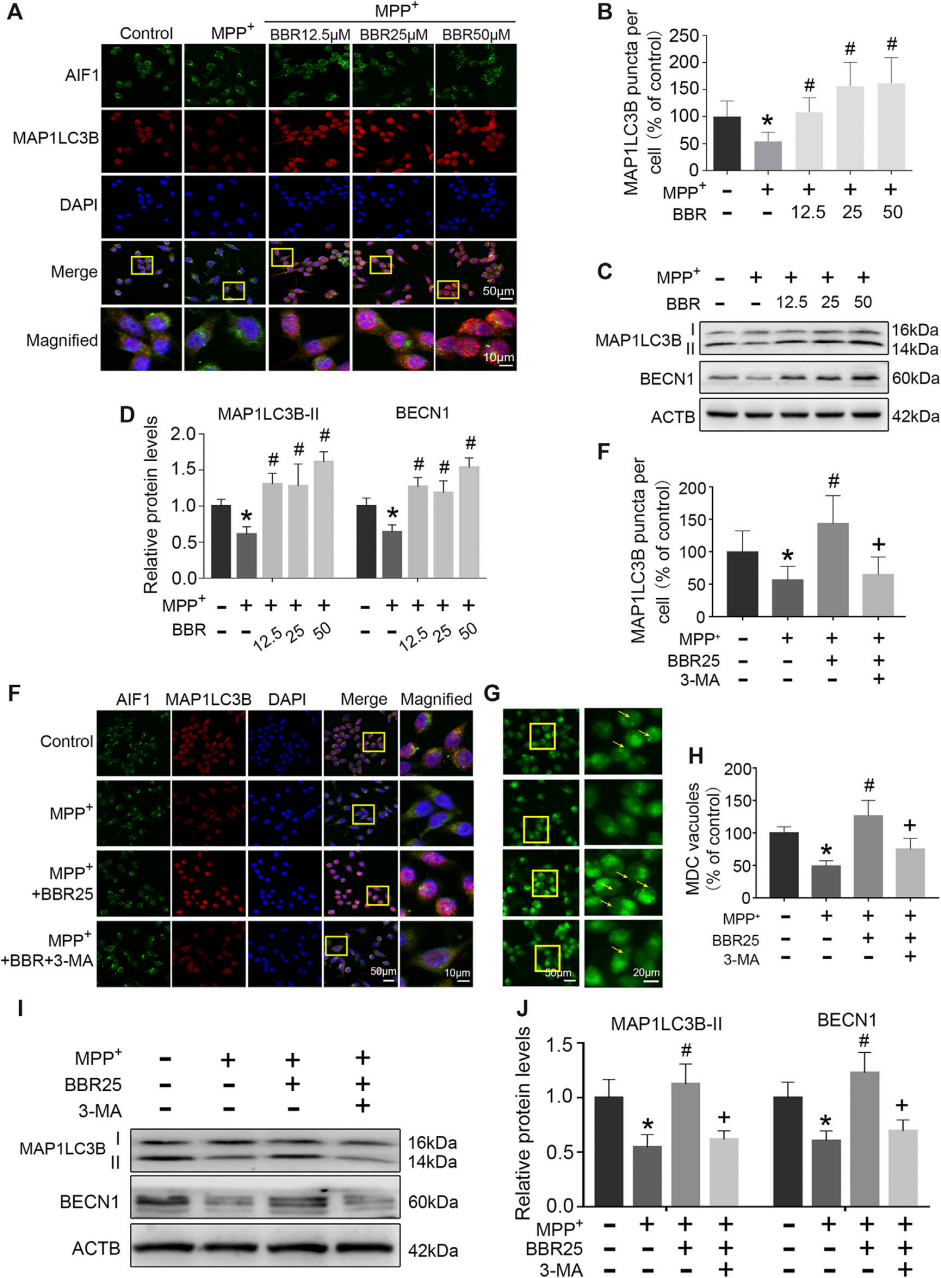
BBR enhances autophagic activity in MPP^+^-treated BV2 cells. The representative immunofluorescent staining **(A)** and puncta **(B)** of MAP1LC3B in BV2 cells treated with MPP^+^ at 200 μM and BBR at the concentration of 12.5, 25, and 50 μM (20 cells were analyzed per group for MAP1LC3B puncta counting). Representative western blots **(C)** and the statistical graph **(D)** of MAP1LC3B and BECN1 in BV2 cells treated with MPP^+^ at 200 μM and BBR at the concentration of 12.5, 25, and 50 μM. The representative double-immunofluorescent staining **(E)** and puncta **(E)** of MAP1LC3B in BV2 cells treated with MPP^+^ at 200 μM, BBR at 25 μM and 3-MA at 10 mM (20 cells were analyzed per group for MAP1LC3B puncta counting). The representative monodansylcadaverine staining **(G)** and statistical graph **(H)** of autophagic vesicles in BV2 cells treated with MPP^+^ at 200 μM, BBR at 25 μM and 3-MA at 10 mM. Representative western blot bands **(I)** and the statistical graph **(J)** of MAP1LC3B and BECN1 in BV2 cells treated with MPP^+^ at 200 μM, BBR at 25 μM and 3-MA at 10 mM. Data were expressed as the mean ± SD (*n* = 3). *p < 0.05 compared with untreated group, ^#^ *p* < 0.05 compared with MPP^+^ group, ^+^ *p* < 0.05 compared with MPP^+^ + BBR group. MAP1LC3B, microtubule associated protein 1 light chain 3 beta; BECN1, beclin 1.

The representative immunofluorescent images and statistical graphs of MAP1LC3B showed that MPP+ at 200 μM significantly decreased the positive cells and puncta of MAP1LC3B in BV2 cells (*p* < 0.05), which was increased by BBR at the concentrations of 12.5, 25, and 50 μM (all *p* < 0.05, Figures 7A,B). Compared to untreated group, MPP+ at 200 μM significantly impaired autophagic activity with a decrease in the expression of BECN1 and MAP1LC3B-II in BV2 cells (both *p* < 0.05, Figures 7C,D), which were significantly increased by BBR at the concentration of 12.5, 25, and 50 μM (all *p* < 0.05, Figures 7C,D).

Additionally, immunofluorescence staining results showed that 3-MA could reduce the positive cells and puncta of MAP1LC3B in BV2 cells when treated with MPP+ at 200 μM plus BBR at 25 μM (*p* < 0.05, Figures 7E,F). Compared to untreated group, MPP+ at 200 μM reduced the formation of autophagic vesicles in BV2 cells (*p* < 0.05), which was increased by BBR at 25 μM (*p* < 0.05, Figures 7G,H). While 3-MA decreased the formation of autophagic vesicles when BV2 cells were co-treated with MPP+ (200 μM) and BBR (25 μM) (*p* < 0.05, Figures 7G,H). We further observed that, 3-MA significantly blocked autophagic activity with a decrease in the expression of BECN1 and MAP1LC3B-II in BV2 cells when treated with MPP+ (200 μM) and BBR (25 μM) (both *p* < 0.05, Figures 7I,J). Besides, 3-MA could also reverse the expressions of NLRP3, PYCARD, cleaved CASP1, and mature IL1B upon co-treated with MPP+ and BBR as shown in Supplementary Figure S2.

## Discussion

As a housekeeping pathway and mediator of cellular homeostasis, autophagy plays an essential role in regulating the activation of NLRP3 inflammasome (Liu et al., 2020a). Studies have shown that autophagy process is impaired during neuroinflammation (Du et al., 2017; Wang et al., 2017; Ali et al., 2020). Reversing autophagic dysfunction which can block NLRP3 inflammasome may provide a novel therapy against PD (Haque et al., 2020). It was reported that BBR prevents DA neuron from death in SN of MPTP-induced mice (Friedemann et al., 2016), but the underlying mechanism remains unclear. Here our data revealed that BBR ameliorated MPTP/MPP+-induced neurotoxicity by enhancing autophagy activity and inhibiting the activation of NLRP3 inflammasome. Besides, inhibition of autophagy with 3-MA antagonized the neuroprotective effects of BBR on MPTP/MPP+-induced neurotoxicity by activating NLRP3 inflammasome. Taken together, our data revealed that BBR could inhibit the activation of NLRP3 inflammasome by enhancing autophagic functions in PD models.

The NLRP3 inflammasome is a multi-protein complex consisting of NLRP3, PYCARD adaptor and CASP1 (Swanson et al., 2019). Chronic activation of microglia is a characteristic of neuroinflammation in PD (Panicker et al., 2019), which closely relates to the activation of NLRP3 inflammasome (Nizami et al., 2019). Environmental toxins such as MPTP, rotenone, 6-hydroxydopamine (6-OHDA) or lipopolysaccharide (LPS) were reported to activate NLRP3 inflammasome in microglia and cause DA neuronal death (Zhou et al., 2016; Mao et al., 2017; Haque et al., 2020). In this study, we found that MPTP induced the activation of NLRP3 inflammasome in microglia which led to DA neuron degeneration and behavior dysfunction in mice. Additionally, in line with previous studies (Yao et al., 2019; Zhang et al., 2020), we observed that MPP+ significantly activated NLRP3 inflammasome by increasing the levels of NLRP3, PYCARD, cleaved CASP1, and mature IL1B. The mechanism of MPP+ activating NLRP3 inflammasome may be explained by that MPP+ stimulates superabundant generation of reactive oxygen species (ROS), which considers as the primary mechanism to activate NLRP3 inflammasome (Groß et al., 2016). Overproduction of ROS dissociates thioredoxin interacting protein (TXNIP), and this dissociation of TXNIP activates NLRP3 inflammasome by directly binding to NLRP3 (Kim et al., 2014; Heo et al., 2019). As a result, the activation of NLRP3 inflammasome releases cleaved CASP1 and also cleaves pro-IL1B and pro-IL18 into mature IL1B and mature IL18, triggering inflammatory cascades (Wang et al., 2019) and causing synuclein alpha (SNCA) aggregation in PD (Wang et al., 2016).

Autophagy eliminates damaged organelles, misfolded proteins and stress-related products to maintain cellular homeostasis (Peker and Gozuacik, 2020). During the process of autophagy, MAP1LC3B-I can be conjugated to phosphatidylethanolamine by cysteine proteases and converted into MAP1LC3B-II (Galluzzi et al., 2017; Ruan et al., 2020). The expression of MAP1LC3B-II is widely used to estimate the autophagic activity (Kim et al., 2017; Yuan at al., 2017). BECN1, known as ATG6 or VPS30, regulates lipid kinase vps34 to promote the formation of BECN1-vps34-vps15 complex and initiates the formation of autophagosome (Levine and Kroemer, 2008; Kang et al., 2011). As a crucial molecule, BECN1 is usually used to monitor autophagic activity (Sun et al., 2018b). It was reported that autophagy activity was impaired in both MPTP-induced mice and MPP+-treated cells (Sun et al., 2018a; Chen et al., 2019; Lin et al., 2020). Consistently, we found that MPTP or MPP+ impaired autophagic activity by decreasing MAP1LC3B-II and BECN1 expression, along with reducing the formation of autophagosomes. Previous studies have confirmed that autophagic activity is tightly linked to the activation of NLRP3 inflammasome (Han et al., 2019; Houtman et al., 2019). Enhancing autophagic activity could inhibit NLRP3 inflammasome activation by removal of damaged mitochondria and prevention of ROS release into cytoplasm (Iida et al., 2018; Liu, 2019). On the contrary, inhibition of autophagy and/or lysosome functions may lead to the activation of NLRP3 inflammasome. One recent study reported that autophagy inhibitor 3-MA and lysosome inhibitor chloroquine (CQ) could enhance the activation of the NLRP3 inflammasome in a rat model of chronic cerebral hypoperfusion (Su et al., 2019). Moreover, 3-MA was recently reported to activate NLRP3 inflammasome in influenza virus-infected macrophages (Liu et al., 2020b). CQ could abrogate the inhibitory effect of metformin on NLRP3 expression in a mouse model of acute myocardial infarction (Fei et al., 2020). Thus, induction of autophagy to suppress the activation of NLRP3 inflammasome is important to against NLRP3-associated disorders (Wang et al., 2020). In this study, we found that NLRP3 inflammasome was activated in both MPTP-induced mice and MPP+-treated BV2 cells which accompanied by the impaired autophagy, indicating the activation of NLRP3 inflammasome may ascribe to the autophagy impairment. Additionally, the autophagic flux is the complete process of autophagy, in which the autophagosomes are lysed by lysosomes. It has been found that BBR could activate the autophagic flux process under several pathological conditions such as cholesterol-overloaded liver (Sun et al., 2018), and induced autophagy flux in myocardial tissue in hypoxia/reoxygenation injury (Zhu et al., 2020). Hence, we speculate that BBR may influence the autophagic flux in PD development according to our present results and literature reports, with which further study needs to be verified.

BBR exhibits several protective effects on neural cells (Song et al., 2020), but little to know of its potential mechanisms in PD. In our data, BBR suppressed NLRP3 inflammasome and enhanced autophagic activity in both MPTP-induced mice and MPP+-treated BV2 cells, indicating that NLRP3 inflammasome may be a target of BBR on inhibiting neuroinflammation. It was reported that BBR displays multiple pharmacological effects on modifying autophagy (Song et al., 2020). Zhang et al. demonstrated that BBR enhanced autophagic activity by promoting autophagosome formation and increasing the expression of BECN1 and MAP1LC3B-II (Zhang et al., 2016). In APP/tau/PS1 mouse, BBR promoted autophagic clearance of amyloid *β* (Aβ) by enhancing autophagic activity through the class-III phosphoinositide 3-kinase (PI3K)/BECN1 pathway (Huang et al., 2017). Zhou et al. reported a novel mechanism of BBR in protecting insulin resistance by enhancing autophagy to inhibit the activation of NLRP3 inflammasome (Zhou et al., 2017). In our experiments, BBR significantly suppressed the activation of NLRP3 inflammasome and enhanced autophagic activity in PD models. Furthermore, we used the common autophagy inhibitor 3-MA to inhibit autophagy to identify whether the inhibition of autophagy causes the activation of NLRP3 inflammasome in PD. As an autophagic inhibitor, 3-MA inhibits autophagy at the early stage of autophagosome formation by inhibiting class-III PI3K (Zeng et al., 2012). Studies reported that 3-MA inhibited autophagy, reduced the expression of MAP1LC3B-II and caused neuronal death (Zhong et al., 2019; Guo et al., 2020). In the present study, we found that the pharmacological effects of BBR were abolished by 3-MA co-treatment, indicating the mechanism of BBR on inhibiting NLRP3 inflammasome may ascribe to the enhancement of autophagy. Moreover, study has been reported that BBR displayed weak effect on the pro-IL1B processing in lipopolysaccharide plus palmitate induced bone marrow derived macrophages (Zhou et al., 2017). Enhancing autophagy could promote NLRP3 autophagic degradation to inhibit NLRP3 inflammasome (Han et al., 2019), indicating BBR may inhibit NLRP3 inflammasome by increasing NLRP3 autophagic degradation.

## Conclusions

In this study, we revealed that BBR could ameliorate PD-like pathophysiology by enhancing autophagy process and inhibit the activation of NLRP3 inflammasome, which provides a novel neuroprotective mechanism of BBR and to be a potential therapeutic agent for PD.

## DATA AVAILABILITY STATEMENT

The raw data supporting the conclusion of this article will be made available by the authors, without undue reservation.

## ETHICS STATEMENT

The animal study was reviewed and approved by the institutional animal care committee of Guangzhou Medical University.

## Author Contributions

SH, LL, and HL designed the research. YWL, ML, and YHL performed the cellular experiments. SH, HM, ZZ, YZ, and PY performed the animal experiments. SH, LD, ZZ, and XH analyzed all experimental data. SH, LL, XY, CC, and XZ drafted and revised the manuscript. LL, PX, and WG supervised this project and revised the manuscript. All authors read and approved the final manuscript.

## Funding

This work was supported by research grants from National Key R&D Program of China (No. 2016YFC1306601 and 2017YFC1310300), National Natural Science Foundation of China (No. 82071416, 81870992, 81870856, and 81771401), a technology project of Guangzhou (No. 2018-1202-SF-0019 and 2019ZD09).

## Conflict of Interest

The authors declare that the research was conducted in the absence of any commercial or financial relationships that could be construed as a potential conflict of interest.
