# Supplementary figures and images for: Berberine Protects Against NLRP3 Inflammasome via Ameliorating Autophagic Impairment in MPTP-Induced Parkinson’s Disease Model
Source: Front Pharmacol. 2021 Jan 27;11:618787. doi: 10.3389/fphar.2020.618787 (PMC7872967; doi:10.3389/fphar.2020.618787)

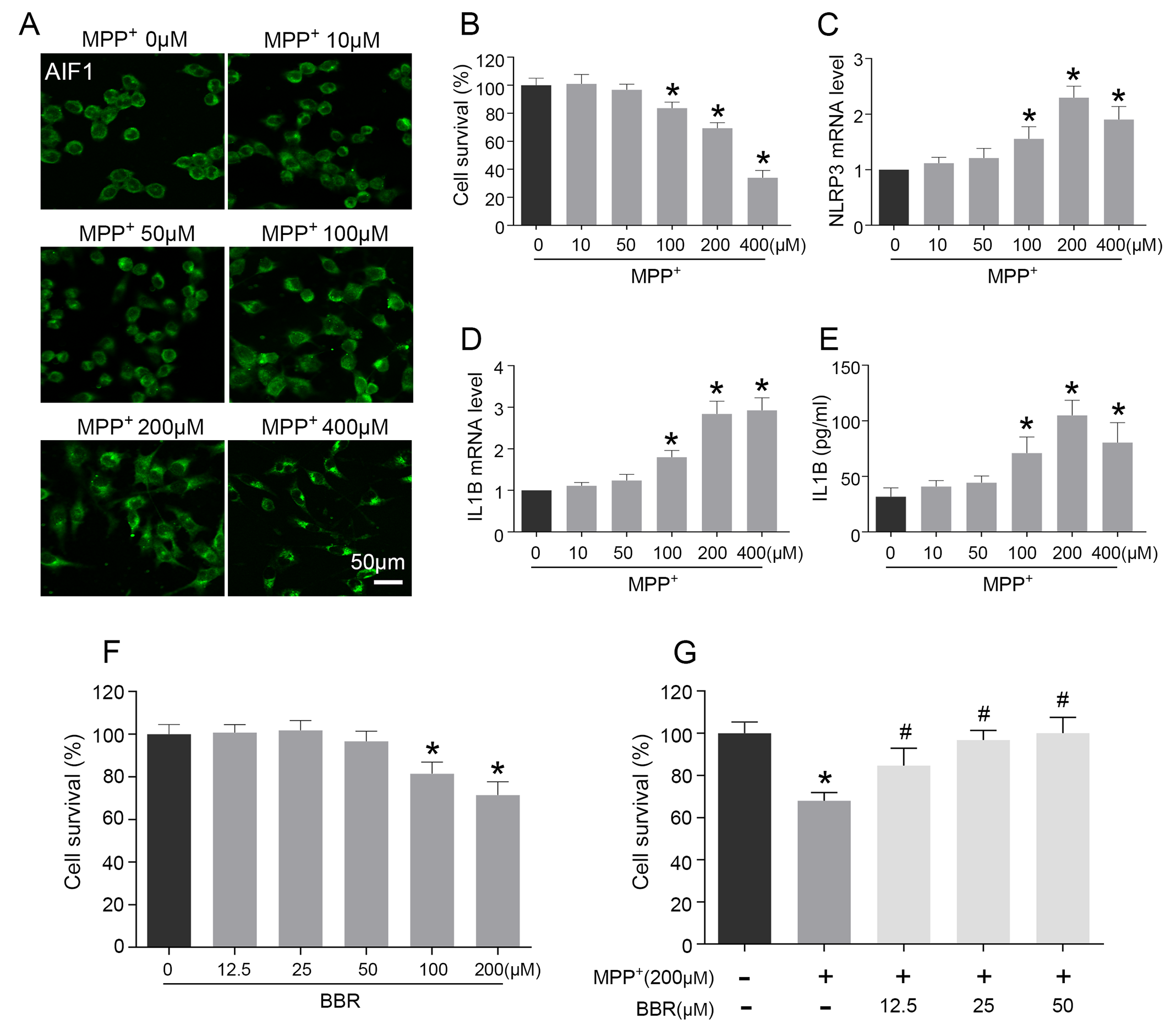

Supplement: Supplementary file 1 [file image1.tif]

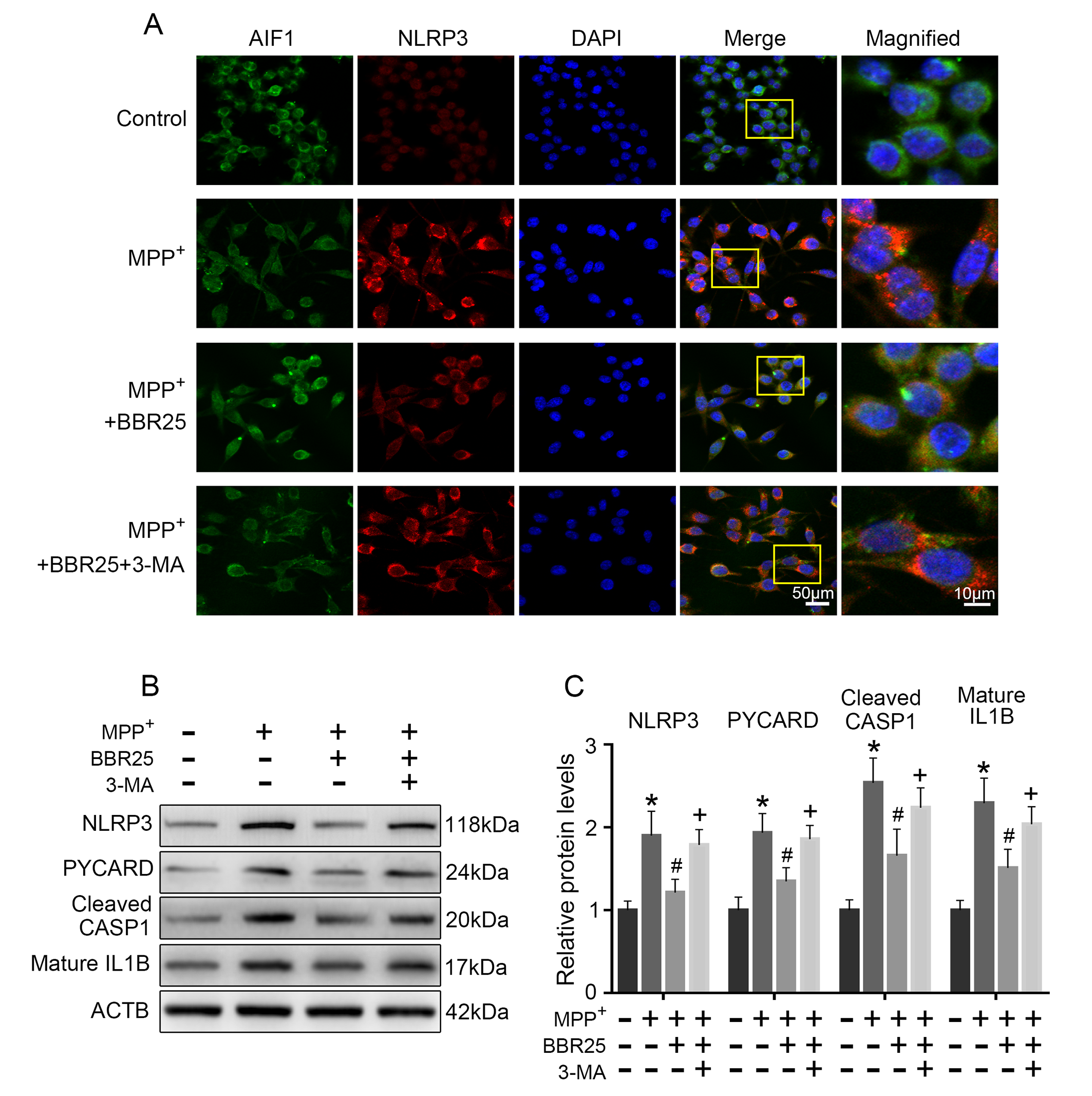

Supplement: Supplementary file 2 [file image2.tif]
